# Global Recognition of Traumatic Brain Injury as a Chronic and Notifiable Condition: A Post-WHA78 Advocacy Commentary

**DOI:** 10.3390/brainsci16020134

**Published:** 2026-01-27

**Authors:** Almas F. Khattak, Saniya Mediratta, Sara Venturini, Brandon George Smith, Paul T. Dubetz, Ernest J. Barthélemy, Alexis F. Turgeon, David Krishna Menon, Bernice G. Gulek, Mario Ganau, Halinder S. Mangat, Kathryn Hendrick, Taskeen Ullah Baber, Yashma Sherwan, Eylem Ocal, Kee B. Park, Walt D. Johnson, Franco Servadei, Gail Rosseau, Peter J. A. Hutchinson, Tariq Khan

**Affiliations:** 1 Global Alliance for Surgical, Obstetric, Trauma and Aneshthesia Care (G4 Alliance) and Community Medicine, Northwest School of Medicine, Peshawar 25000, Pakistan; almasfasih@gmail.com; 2 Department of Neurosurgery, National Hospital for Neurology and Neurosurgery, London WC1N3BG, UK; 3 NIHR Global Health Research Group on Acquired Brain and Spine Injury, University of Cambridge, Cambridge CB20QQ, UK; sv465@cam.ac.uk (S.V.); pjah2@cam.ac.uk (P.J.A.H.); 4 Department of Engineering, University of Cambridge, Cambridge CB2 1PZ, UK; bgs30@cam.ac.uk; 5 William G. Lowrie Department of Chemical & Biomolecular Engineering, The Ohio State University, Columbus, OH 43210, USA; dubetz.2@osu.edu; 6 One Brooklyn Health, Brookdale University Medical Center, Brooklyn, NY 11212, USA; globalneurosurgeon@gmail.com; 7 SUNY Downstate Health Sciences University, Brooklyn, NY 11203, USA; 8 Population Health and Optimal Health Practices Research Unit (Trauma-Emergency-Critical Care Medicine), CHU de Québec—Université Laval Research Center, 1401, 18e Rue, Québec City, QC G1J 1Z4, Canada; alexis.turgeon@crchudequebec.ulaval.ca; 9 Division of Critical Care Medicine, Department of Anesthesiology and Critical Care Medicine, Faculty of Medicine, Pavillon Ferdinand-Vandry, 1050 Av. de la Médecine, Université Laval, Québec City, QC G1V 0A6, Canada; 10 Department of Medicine, University of Cambridge, Cambridge CB2 0AW, UK; dkm13@cam.ac.uk; 11 Harborview Medical Center, Department of Neurological Surgery, University of Washington, Seattle, WA 98104, USA; ggulek@gmail.com; 12 School of Medicine, BAU International University Batumi, 237 Fridon Khalvashi St., 6010 Batumi, Georgia; mario.ganau@alumni.harvard.edu; 13 Department of Neurology, Weill Cornell Medical College, New York, NY 10021, USA; hsm9001@med.cornell.edu; 14 Department of Neurology, Kansas University Medical Center, Kansas City, KS 66160, USA; 15 Canadian Traumatic Brain Injury Research Consortium, CHU de Québec—Université Laval (Hôpital de l’Enfant-Jésus) 1401 18e Rue, Québec City, QC G1J 1Z4, Canada; hendrickkathryn@gmail.com; 16 Global Surgery Regional Hub, Northwest School of Medicine, Peshawar 25000, Pakistan; taskeenbaber@gmail.com (T.U.B.); yashmasherwan8@gmail.com (Y.S.); 17 Department of Neurosurgery, University of Arkansas for Medical Sciences, Little Rock, AR 72205, USA; eocal@uams.edu; 18 Arkansas Children’s Hospital (ACH), Little Rock, AR 72223, USA; 19 Program in Global Surgery and Social Change, Harvard Medical School, 641 Huntington Avenue, Boston, MA 02115, USA; keepark@yahoo.com; 20 Department of Neurosurgery, Loma Linda University, 12354 Anderson Street, Loma Linda, CA 92354, USA; 21 Global Neurosurgery Programme Fondazione IRCCS Istituto Neurologico Carlo Besta, Via Giovanni Celoria 11, 20133 Milan, Italy; francoservadei@gmail.com; 22 Department of Neurosurgery, George Washington University, Washington, DC 20052, USA; gailrosseaumd@gmail.com; 23 Department of Neurosurgery, Northwest General Hospital & Research Centre, Peshawar 25000, Pakistan

**Keywords:** traumatic brain injury (TBI), notifiable condition, chronic disease, lived experience, rehabilitation, surveillance, world health assembly, WHA resolution, global health policy, the Global Coalition for TBI

## Abstract

Background: Traumatic brain injury (TBI) is a leading cause of disability but one of the least recognized health problems in the world, affecting up to 69 million people annually. The associated lifelong disability in survivors, the loss of economic productivity, and being a risk factor for dementia consume 0.5% of global economic activity. Yet TBI is still largely invisible in national surveillance systems and not well represented in chronic disease frameworks. Consequently, governments are not equipped to provide proportional financing of acute care and long-term care of survivors, nor to build health care systems and resources for improving outcomes of TBI through policy frameworks targeting prevention, treatment, and equitable access. Objective: This commentary aims to provide a comprehensive picture of the global effort to formally recognize TBI as a notifiable and chronic condition, including the justifications for recognition, the formation of an international coalition of stakeholders, and the strategic plan for resolution at WHA79 of the World Health Assembly, one of the first concerted multinational efforts that occurred as a side event during the 78th World Health Assembly (WHA78) in May 2025. Methods: This commentary integrates information from epidemiological studies, global registries, and testimonies from people with lived experience of TBI. We analyze these data to develop policy needs and corresponding initiatives to address key needs. These include coordinated efforts to advocate change, such as technical briefings, consultations with stakeholders, and storytelling led by survivors, all of which informed and formed a part of the WHA78 side event. Our efforts have garnered wide, multi-sector support. Results: The WHA78 side event showed that ministries of health, neurosurgical, neurological, and rehabilitation societies, academic researchers, WHO representatives, and survivors all unprecedentedly support the recognition of the importance of TBI, facilitating national policies for its prevention and treatment via standardized surveillance. More than 30 non-governmental groups officially supported the campaign. A sponsoring member state made a public commitment to co-sponsor a WHA resolution, which set the stage for ongoing diplomatic progress and engagement across regions. Conclusion: To improve global brain health equity, access to long-term care, and the resilience of health systems, it is important to recognize TBI as a notifiable and chronic condition. A dedicated WHA resolution would make TBI a part of global health governance, making sure that it is counted, tracked, and dealt with as quickly and comprehensively as possible. It is both a technical necessity and a moral duty to help survivors and families and fight for justice in global health systems.

## 1. Background on WHA

The World Health Assembly (WHA) is the highest decision-making body of the World Health Organization (WHO), with 194 member states. Every year, delegates from all member states meet at the WHA following a carefully planned agenda specified by the WHO Executive Board to set the direction for global health policy. It oversees the organization’s goals, approves its budget, oversees its finances, and appoints the Director-General [1].

With the theme “One World for Health”, the 78th World Health Assembly (WHA78) convened from May 19th to the 27th, 2025, to represent international unity and a renewed commitment to multilateralism in health policymaking. Technical briefings, side events, and ministerial roundtables enhanced the Assembly’s activities [2]

One key achievement of WHA78 was the adoption of the Pandemic Agreement, and the Ministerial Roundtable at the UN Palais des Nations focused on how nations can use improved health data and long-term funding to strengthen their health care systems and achieve the Sustainable Development Goals and universal health coverage [3,4,5].

WHA78 adopted a number of important resolutions in addition to the Pandemic Agreement, such as substandard and falsified medical products (WHA78.12), antimicrobial resistance (WHA78.15), the global strategy on digital health 2020–2025: extension (WHA78.22), and the global action plan on climate change and health (WHA78.27) [6].

To address pressing issues like global surgery, rehabilitation, and mental health integration, more than 75 agenda items were formally considered, and several technical briefings were held. Additionally, WHA78 was a significant gathering place for academic institutions, public health networks, and civil society, leading to numerous high-level multi-disciplinary side events [2,7].

The Global Coalition for TBI (Supplementary content)hosted a side event titled “Recognizing Traumatic Brain Injury (TBI) as a Notifiable and Chronic Condition”, which supported the WHA78 theme of improving health systems through inclusive, evidence-based policy responses. The event aimed to position TBI as a critical component of neurological, trauma, and rehabilitation care agendas, in line with the WHO’s Global Brain Health Strategy and the Rehabilitation 2030 initiative [8,9].

## 2. Introduction

Traumatic brain injury (TBI) remains one of the most urgently pressing yet underrecognized global health issues. TBI affects an estimated 69 million people globally each year, with low- and middle-income countries (LMICs) bearing a disproportionately heavy component of the burden due to increased rates of road traffic accidents, falls, and violence [10]. According to the 2019 Global Burden of Disease Study, TBI is one of the leading causes of years lived with disability (YLDs), particularly for working-age adults and adolescents, where injuries like traffic accidents and interpersonal violence continue to be the main causes of disability-adjusted life years (DALYs) [11]. Despite this significant influence, TBI is still primarily viewed as an acute, episodic condition that is inconsistently diagnosed and frequently disregarded in international policy and public health initiatives. The ongoing discrepancy between the actual burden of TBI and its acknowledgement in global health governance is the root cause of the lack of consistent visibility in surveillance systems and national health agendas [12]. In addition to being a matter of health equity, closing the gap in TBI care is strategically necessary to lower long-term disability, financial loss, and societal burden on a worldwide scale [13].

TBI is becoming more widely acknowledged as a chronic, life-changing condition that often has long-lasting effects, rather than as a single, acute episode. Survivors commonly endure new and worsening neuropsychiatric disorders, functional limitations, and long-lasting cognitive impairments that go well beyond the initial phase of recovery [14]. According to the evidence, people with TBI may experience a decline in neurological function, emotional control, and general quality of life even years after the injury. They may also be at higher risk of developing epilepsy, stroke, and neurodegenerative illnesses like Alzheimer’s disease and related dementias or Parkinson’s disease. Recently, an influential Lancet Commission on Dementia recognized TBI as a key preventable risk factor for dementia [15]. The magnitude of this long-term burden is evident in the fact that, in 2014, TBI was associated with nearly 2.87 million emergency room visits, hospitalizations, or deaths in the United States alone [16]. It is important to note that the effects of TBI vary across the population. For example, older adults are more likely to experience unfavorable outcomes and have higher mortality because of pre-existing medical conditions adding to the burden of TBI. This functional decline frequently persists for a long time after the initial injury, supporting the notion that TBI is a chronic condition requiring ongoing care, monitoring, and policy-level attention [17].

In most national health surveillance systems, TBI is largely invisible, despite the substantial burden it poses. When considered, TBI is often viewed like any other traumatic injury: something you either recover from or do not survive. Because TBI is not listed as a chronic condition or on the list of diseases that require notification in most countries, data collection is dispersed, long-term follow-up is subpar, and public health accountability is restricted [18]. This lack of visibility in data and policy not only makes it difficult to accurately estimate the burden but also jeopardizes long-term care financing, strategic planning, and service delivery. TBI and other neurological conditions are underreported and underserved in health systems worldwide. This is even more important in LMICs where access to neurological services and data systems is still limited, according to the WHO report on neurological disorders [19,20]. Aiming to systematically collect high-quality, anonymized data across settings and income levels, programs such as the Global Epidemiology and Outcomes following Traumatic Brain Injury (GEO-TBI) registry have emerged to fill these gaps [21]. For data governance, benchmarking, and research in a variety of international contexts, GEO-TBI provides a cooperative model. However, there may be regulatory barriers to collecting data in such international initiatives that will only be supplemental rather than revolutionary until nations formally recognize TBI in their national reporting frameworks and chronic disease registries. Closing the data gap is not just a technical problem; it is essential to long-term health system resilience, equity, and policy visibility.

The 78th World Health Assembly (WHA78) in May 2025 marked a significant milestone in global neurotrauma advocacy. It served as a launching pad for a collective international call to recognize TBI as a notifiable and chronic condition. The Global Coalition for TBI organized a momentous event on the sidelines of WHA78, titled “Recognizing TBI as a Chronic and Notifiable Condition”, attended by international organizations, global health care leaders, ministries of health, athletes, researchers, and patient advocates, highlighting the need for integrated data systems, rehabilitation pathways, and policy reform to address TBI. This global effort builds on existing WHO frameworks and Sustainable Development Goal 3’s focus on universal health coverage (UHC). The WHA78 advocacy effort also strengthened equity in future pandemic preparedness and chronic disease policy, emphasizing the importance of data-driven priorities like TBI [22].

This commentary summarizes the WHA78 side event on TBI and its significance as a launchpad for global policy action. In addition to outlining the collaborative efforts that shaped this campaign, it provides a roadmap for a formal resolution at WHA79 and explains the rationale behind the call to recognize TBI as a chronic and notifiable condition. The initiative, which draws from registry data, national strategies, patient testimonies, and technical inputs, reflects a growing consensus that TBI’s invisibility in global health governance can no longer be ignored. Health ministries, academic institutions, neurosurgical and neurological societies, athletic representatives, and advocacy groups are among the diverse coalition of international partners spearheading the campaign. These partners are united in their commitment to patient-centered care, data transparency, and health equity. This commentary supports global public health priorities and a larger movement advocating evidence-based, inclusive, and equitable health systems by placing TBI within the broader framework of UHC, disability rights, and brain health. The momentum created at WHA78 offers a strategic opportunity and a moral imperative to reframe TBI as a global health priority that cannot be disregarded as the world moves toward WHA79.

This commentary uses the WHA78 event as a policy catalyst to advocate global recognition of TBI as a notifiable, chronic, and preventable condition and to outline a roadmap towards a standalone resolution by the WHA.

## 3. Why the Need for TBI to Be Recognized as a Notifiable and Chronic Condition?

Despite growing evidence of long-term effects, TBI, which affects millions of people worldwide each year, is rarely classified as more than an acute injury fixed in time. Long into their recovery period, survivors struggle with persistent and changing conditions that last for years or even decades, such as fatigue, emotional instability, functional deficits, chronic cognitive impairment, and up to full dependence on others [14,23]. For instance, there is evidence that mild TBI could increase the risk of Parkinson’s disease by more than 50%, while moderate-to-severe TBI has been linked to nearly doubling the risk of dementia (90% increased risk) [24,25]. These long-lasting effects, among others, highlight why TBI is a chronic lifelong illness rather than a temporary illness.

The widespread underreporting and lack of visibility of TBI in public health surveillance systems exacerbate this problem. Both TBI incidence and prevalence are consistently undercounted because estimates are frequently based only on hospital admissions or death records, ignoring minor injuries not thought to require urgent care or unreported injuries in countries with no universal health care system coverage or limited health care resources. Symptoms later in life may thus not always be linked with the TBI that occurred months or years before. Countries lack the comprehensive data required to influence policy, allocate resources, or measure outcomes because TBI-related emergency visits, hospitalizations, and deaths are routinely characterized by global injury data without consistent notifiability requirements. Since TBI is not officially recognized as a chronic and notifiable condition, it is not included in national health strategies, rehabilitation initiatives, or social support programs, leading to the invisibility of TBI. Furthermore, while the causes, incidence, and prevalence of TBI vary throughout the world, the overall burden is expected to increase, particularly among vulnerable populations, because of ongoing conflicts, climate-related disasters, urbanization, and demographic shifts [26]. By formally recognizing TBI as both a notifiable and chronic condition, it would help countries support inclusive disability agendas, UHC, and emergency preparedness, while also aligning their health policies with Sustainable Development Goal 3 (ensuring health and well-being for all) [27].

Transforming disparate and incomplete information into more complete and integrated data would support the development of precise, actionable reforms, encourage timely rehabilitation, and guarantee lifelong care for millions of survivors. Formally integrating such key data regarding TBI into national reporting and chronic disease registries would have important policy consequences: what is counted gets funded, and what gets funded is fixed.

## 4. Inception of the Global Coalition for TBI

An initiative from the Canadian Traumatic Brain Injury Consortium and Brain Injury Canada in 2022 led to the dissemination of a position paper to recognize TBI as a chronic condition [28]. This initiative led to meetings with international colleagues, clinicians, and researchers in the field of TBI, aiming to form a global coalition to achieve this overarching objective. At a meeting in Cambridge in 2023, a shared concern and a united resolve gave rise to the beginning of a Global Coalition for the Recognition of TBI as a Chronic and Notifiable Condition. This gathering, which was centered around the NIHR Global Health Research Group on Acquired Brain and Spinal Injury (ABSI) initiative’s launch, brought together neurosurgeons, intensivists, researchers, policy experts, rehabilitation specialists, and community advocates. What started out as a discussion about the lack of awareness, research, and care for brain injury in LMICs swiftly turned into a collective call for TBI to be recognized globally as a chronic, notifiable condition that requires long-term surveillance, funding, and policy changes.

The Global Coalition for TBI quickly grew in the months that followed, encompassing more than 35 institutions and groups from various disciplines, sectors, and continents. A unique combination of academic institutions, trauma centers, neurosurgical societies, emergency and critical care networks, associations for brain injuries, rehabilitation networks, community-based organizations, patient advocates, and technical partners from all parts of the globe make up the Coalition. Members are bound together by a common dedication to the values of equity, data justice, and lived experience, despite differences in geography and institutions. When taken as a whole, the Coalition acknowledges that TBI is not just a medical condition but also a profoundly political, social, and economic issue that is mostly ignored by public health systems.

The Coalition has approached its work from the perspective of long-term system change since its inception. In addition to supporting community-based rehabilitation, survivor inclusion in policy design, and the creation of straightforward, scalable notification tools for both high- and low-resource settings, it pushes for the inclusion of TBI in national notifiable disease lists and chronic disease registries. The Coalition functions as a strategic alliance, bridging the technical know-how of researchers and health experts with the grassroots urgency of patient-led advocacy, in contrast to issue-specific advocacy groups.

Despite the relatively short time since its inception, the Coalition had a catalytic effect. It organized the first wave of international institutional support for a WHA resolution, set the stage for the Geneva WHA78 side event, and served as the foundation for diplomatic relations with several WHO member states. Its resources, which include a position paper, opinion editorial, and testimonies from survivors, are being disseminated among UN agencies, global consortia, and health ministries, strengthening the narrative and supporting data for policy action. As a result, what was formerly a loosely affiliated network has evolved into a coordinated, action-oriented campaign with a well-defined objective: to obtain a resolution at the 79th World Health Assembly that would formally recognize TBI as a chronic and notifiable condition.

By elevating the voices of survivors, organizing health systems, and reorienting global health priorities toward long-overdue accountability for brain injury, the Global TBI Coalition is still growing today. By doing this, it embodies both a technical movement and a moral obligation to care for those who have been left behind and to count what has long gone uncounted.

The Coalition has actively engaged WHO regional offices and health ministries in several nations to further its goal of formal recognition for TBI. The Coalition is attempting to obtain political commitment for co-sponsorship of the proposed WHA79 resolution through bilateral consultations, targeted advocacy briefings, and the distribution of technical materials. These interactions highlight the necessity of closing a long-standing policy gap, the viability of TBI reportability, and its alignment with current WHO strategies. The WHA78 side event in Geneva, a significant moment in the campaign, was made possible by these combined efforts.

## 5. WHA78 Side Event Overview

The side event at the WHA78 titled “Recognizing TBI as a Chronic and Notifiable Condition”, which took place on 21 May 2025, was a turning point in the global campaign for TBI advocacy and policy reform. The event is presented here not as a detailed meeting report, but as a synthesis of key advocacy-relevant themes that informed the global policy call to recognize TBI as a notifiable, chronic, and preventable condition.

To strengthen the case for formal recognition of TBI within the global health framework, the Global Coalition for TBI organized this high-level event on the sidelines of the 78th World Health Assembly. Participants included researchers, WHO officials, policymakers, neurosurgeons, allied neurological and non-neurological clinicians, emergency physicians, intensivists, civil society, and survivor advocates.

A representative of the G4 Alliance opened the program by framing the conversation within the larger goals of trauma advocacy and global surgery. To bridge the agendas of surgery, critical care medicine, neurology, and rehabilitation, the speaker underlined the critical need for equity-driven recognition of TBI as a chronic and notifiable condition. A compelling video testimony from a TBI survivor in a setting with limited resources came next. The personal narrative brought attention to the long-term, multifaceted effects of TBI on people and families while also highlighting the effects of policy neglect and inadequate post-discharge care.

A TBI survivor from the corporate world gave an in-person presentation on a second lived experience. The discussion highlighted the challenges of managing long-term recovery, reintegration into the workforce, and the emotional toll on caregivers, all of which were based on personal experience. This compelling story reaffirmed how urgent it is to raise awareness of TBI and the necessity of ongoing, organized rehabilitation programs.

An academic in global health presented next, laying the epidemiological and scientific groundwork for TBI recognition as a notifiable condition. The speaker discussed how TBI disproportionately impacts marginalized communities and young adults, leading to lost productivity, economic exclusion, and long-term disability. According to the presentation, a WHA resolution is a necessary policy intervention to institutionalize accountability and resource mobilization because the burden of TBI remains invisible in the absence of adequate surveillance systems and long-term support infrastructure.

The WHO Brain Health Unit representative then contextualized TBI in relation to the Rehabilitation 2030 initiative and the Brain Health Intersectoral Action Plan. The speaker highlighted the necessity of incorporating TBI into neurological care, rehabilitation frameworks, and noncommunicable disease strategies while restating WHO’s commitment to brain health equity. This was an important input to show how a WHA resolution on TBI would complement current WHO initiatives and give member states the technical authority they require to act.

An official from the Ministry of Health Pakistan announced that their country would support a WHA resolution that recognizes TBI as a chronic and notifiable condition. The national burden of TBI, data gaps, and the advantages of notifiability in enhancing rehabilitation services and long-term results were all topics covered by the official. With this declaration, the campaign underwent a diplomatic sea change, moving from advocacy to official policy engagement at the member state level.

A representative of the neurosurgical society then emphasized how many TBI cases could be avoided with better emergency response systems and legislative initiatives like helmet regulations and traffic safety enforcement. The presentation promoted investment in both surgical and rehabilitation infrastructure, especially in LMICs, and connected the development of neurosurgical capacity to the success of long-term care systems.

A representative from both a national TBI research consortium and a national brain injury representatives’ organization in a high-income country presented the efforts made over the last few years to get TBI recognized as a chronic condition and to pass an act to establish a national strategy on brain injuries in a private bill. What has been learnt from collaborating across different sectors, drafting legislation, and how important it is for survivors to share their stories to gain support from Parliament was discussed. These insights showed that legislative action is achievable and can be replicated elsewhere.

Early data from multi-country studies showing high TBI incidence rates and low follow-up rates were provided by researchers affiliated with the GEO-TBI Registry. They emphasized that in the absence of mandatory notification systems, national responses would be insufficient, and data would continue to be fragmented and incomplete. A WHA resolution could mandate improved data architecture worldwide, strengthening program design and surveillance.

Public health professionals, including a neurosurgeon and global surgery advocate, discussed upstream strategies like community-based interventions, trauma system strengthening, and injury prevention. The speaker made the case that without official recognition, TBI prevention is still dispersed, underrecognized, and underfunded, highlighting the importance of effective prevention strategies coupled with policy-level recognition.

A former athlete and TBI survivor who had sustained multiple concussions shared a unique message after the prevention session. Their message emphasized the invisibility of repeated head trauma, the long-term neurological and emotional effects of TBI in high-performance sports, and the urgent need for early surveillance and structured policies. The audience was reminded by the testimony that TBI is a widespread risk that impacts people from all walks of life and is not limited to areas of conflict or trauma.

At a moderated panel discussion that marked the program’s conclusion, participants and speakers discussed the significance of ongoing survivor engagement, regional routes to resolution sponsorship, and next steps. The panelists reaffirmed that evidence and lived experience must both guide the path to WHA79 and emphasized the role of WHO regional offices in coordinating technical and diplomatic support.

The WHA78 side event showed that science, policy, advocacy, and survivor leadership have never been more in step. It was a big change in the global discourse on TBI around the world, from seeing it as a clinical challenge to making it a global policy priority. The event set the stage for the drafting and progress of a standalone WHA resolution on TBI, bringing together all stakeholders under a single call—to count what has been invisible for too long and to care for those who have been left behind for too long.

## 6. Global Endorsements

The Coalition’s strategic advocacy mobilized extensive institutional support for the recognition of TBI as a notifiable and chronic condition prior to and after the WHA78 side event. These organizations endorsed the coalition’s resolve that addressing long-standing gaps in brain injury care and policy requires this kind of recognition. They emphasized that national and international health reporting systems, which presently ignore large segments of the TBI population, would be considerably more accurate if TBI was recognized as a chronic and notifiable condition. Additionally, it would improve survivors’ long-term access to rehabilitation and follow-up care, as they are frequently left without ongoing social and medical support. They also emphasized that official recognition would allow TBI to be systematically incorporated into current WHO strategies, such as the Rehabilitation 2030 initiative, the intersectoral global action plan on epilepsy and other neurological disorders [8], and larger frameworks addressing emergency care and noncommunicable diseases. The diversity and depth of support show the growing agreement that TBI cannot continue to be a burden that is overlooked within health systems.

These endorsements reflect broad geographic representation across multiple WHO regions and span diverse sectors, including clinical and rehabilitation societies, academic institutions, civil society organizations, and patient advocacy groups.

(This commentary is accompanied by a Supplemental File, Global Endorsements to Recognize TBI as a Chronic and Notifiable Condition, that contains a complete list of endorsing organizations.)

## 7. The Next Steps and the Roadmap to WHA79

After the successful event on the sidelines of WHA78 and the significant momentum it created, the Coalition has entered a significant new phase, one that is centered on turning advocacy into policy. The Coalition is stepping up its efforts to achieve a WHA resolution formally recognizing TBI as a notifiable and chronic condition as the 79th World Health Assembly draws near.

Maintaining diplomatic ties with health ministries and permanent missions of the WHO is essential to this stage. The goal of these initiatives is to find and assist member states that are open to sponsoring or co-sponsoring the proposed resolution. With customized briefings emphasizing how TBI recognition fits with national health priorities, disability inclusion, emergency care, and larger commitments to UHC, discussions are currently taking place with delegations from various regions. To localize advocacy efforts and promote communication between national governments and technical experts, coalition representatives are also actively working with WHO regional offices.

Importantly, recognition of traumatic brain injury as a notifiable condition would not imply a uniform implementation model across countries. As with other globally recognized conditions, approaches to notification and surveillance would be expected to vary across low-, middle-, and high-income settings, with phased and context-appropriate integration into existing trauma, rehabilitation, or health information systems. Detailed operational guidance, including infrastructure, workforce, and data governance considerations, would appropriately follow WHA-level recognition through WHO technical processes and country-led adaptation.

The Coalition has finalized drafting the resolution text, and a major milestone has already been achieved as the governments of Pakistan and Türkiye have officially committed to sponsoring the proposed resolution. Moreover, interest has been expressed by the Ministry of Health of Indonesia, Togo, the Central African Republic (CAR), along with continuous diplomatic outreach across WHO regions. Discussions are underway with the Ministries of Health of Norway and Australia. This will be followed by regional meetings, webinars, and high-level discussions to increase the network of support.

Importantly, TBI survivors’ voices will continue to play a key role in this campaign phase. To highlight the long-term effects of TBI and the policy gaps that still exist in its treatment, survivor stories will be presented in future webinars, podcast series, and live events. These stories will act as a powerful reminder of the human cost of inaction and the pressing need for recognition of TBI.

This roadmap collectively constitutes a concerted, calculated effort to influence policy change. By transforming visibility into accountability and momentum into long-lasting policy change, the Coalition is preparing the way for WHA79 to be a pivotal moment in the global response to TBI.

## 8. Why a Standalone Resolution on TBI Recognition Matters?

More than just a significant policy development, a standalone WHA resolution on TBI is a crucial first step in safeguarding the resilience, brain health, and economic prospects of countries. Despite being a unique intersection of trauma, chronic illness, disability, and mental health, TBI is still not well represented in the governance of global health. Current frameworks frequently include TBI under more general headings, hiding its unique requirements and preventing focused investment and policy.

**Figure 1:**
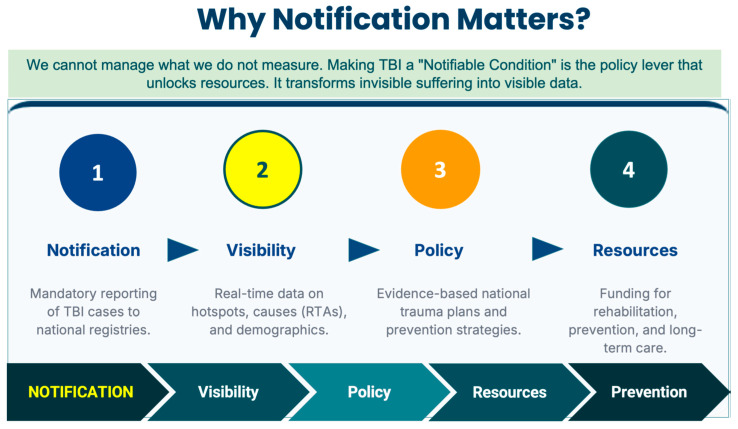
Why notification matters: the data-to-action pathway for traumatic brain injury (TBI).

By establishing a clear mandate for incorporating TBI into national surveillance systems, chronic disease strategies, and UHC agendas, a dedicated resolution would reverse this trend. It would encourage the creation of technical guidelines, formally place TBI within the WHO’s operational priorities, and open new funding avenues for long-term care, prevention, and rehabilitation. Figure 1.

In low- and middle-income countries, initial support pathways for TBI recognition are expected to prioritize feasibility and scalability. This includes the use of simplified, minimum data reporting tools embedded within existing trauma or emergency care records, as well as adaptation of community- and hospital-based rehabilitation models that are already operating in resource-constrained settings. Through its network of academic, clinical, and policy partners, the Global Coalition plans to support LMICs primarily through knowledge sharing, technical advocacy, and alignment with existing WHO emergency care, rehabilitation, and health information system frameworks, rather than introducing parallel systems.

Furthermore, the advancement of the global brain health economy depends on the recognition of TBI through a standalone resolution. TBI undermines national productivity and development because it disproportionately affects young adults, who are the most economically productive demographic. Societies pay high long-term costs in lost educational attainment, labor force participation, and caregiving burden when TBI is not addressed, and opportunities to prevent and mitigate the harmful effects of TBI on healthy aging are neglected. A solution would place TBI in the larger context of brain capital, which refers to the collective social, emotional, and cognitive resources that support learning, innovation, and social cohesiveness in economies of the twenty-first century [29,30,31].

Investing in TBI surveillance, care, and recovery is not only a health necessity but also a socioeconomic strategy in a time when mental health, cognitive resilience, and brain function are increasingly used to define human capital. Given that TBI is estimated to cost the global economy USD 400 billion annually (1 in every USD 200 the world generates), it is a global priority deserving of consistent attention, resources, and accountability through a standalone WHA resolution [12]. This would guarantee that what has long been invisible becomes quantifiable, and what has long been overlooked becomes a foundation for health systems prepared for the future.

## 9. Conclusions

The A78 side event showed that this cause is important to people from all over the world in many different fields. Through a unified call for visibility, equity, and accountability, stakeholders made it clear that TBI must be systematically counted, tracked, and addressed within global health governance.

There is a clear policy, scientific, and moral case for recognition. TBI is a lifelong condition that affects countless young and vulnerable people and has a big impact on their social and economic lives. Nevertheless, it remains largely invisible in many national reporting systems and plans for chronic diseases. A separate resolution from the WHA would make the global response to TBI official. Adoption of a standalone WHA resolution is expected to yield measurable policy outcomes, including the integration of traumatic brain injury into national health surveillance systems and the routine reporting of TBI incidence and outcomes within existing health information platforms. In alignment with Sustainable Development Goal 3, such recognition would support progress toward SDG 3.8 (universal health coverage) and SDG 3.6 (reducing injury-related mortality and disability) by enabling countries to plan, finance, and monitor prevention, acute care, rehabilitation, and long-term support for TBI more systematically.

The road ahead will need more diplomacy, advocacy led by survivors, and technical cooperation, but the foundations for this important work have been laid. The Global Coalition for TBI has built a strong case and secured global support. As we get closer to WHA79, we need to do more than just speak out; we need to act. TBI needs to be recognized with the attention, investment, and respect it deserves, not just ignored. What started as a call to count the uncounted has turned into a movement to care for the long-ignored and to make TBI a visible priority in global health agendas.

Collaborative Authorship: This commentary was prepared through collaborative authorship by representatives and members of the Global Coalition for TBI and WHA78 side event contributors. It reflects a joint consensus of academic, clinical, advocacy, and institutional partners who participated in the event and campaign.

## Data Availability

No new data were created or generated in this study. Data sharing is not applicable.

## Supplementary Materials

The following supporting information can be downloaded at https://www.mdpi.com/article/10.3390/brainsci16020134/s1: Supplementary content: Global Coalition for TBI.
