# Supplementary material for: Global Recognition of Traumatic Brain Injury as a Chronic and Notifiable Condition: A Post-WHA78 Advocacy Commentary
Source: Brain Sci. 2026 Jan 27;16(2):134. doi: 10.3390/brainsci16020134 (PMC12938628; doi:10.3390/brainsci16020134)
Supplement: Supplementary file 1 [file brainsci-16-00134-s001.zip › brainsci-4089714-supplementary.pdf]

Global Coalition for TBI

Endorsing Organisations

The Global Coalition for Traumatic Brain Injury's call to recognise TBI as a notifiable and chronic condition has received formal endorsement from the following organisations.

G4 alliance surgical, obstetric, trauma & anaesthesia care	**G4 Alliance**	G4 Alliance is a coalition of associations and organizations around the world working to increase awareness, foster political will, shape policy, and mobilize resources to make access to quality, safe, timely, and affordable emergency and essential surgical, obstetric, trauma and anaesthesia (SOTA) care a global health priority and a reality for all.
NIHR Global Health Research Group on Acquired Brain and Spine Injuries	**NIHR Global Health Research Group on Acquired Brain and Spine Injuries (ABSI)**	The NIHR Global Health Research Group on Acquired Brain and Spine Injury focuses on five major conditions causing significant disability in low- and middle-income countries: traumatic brain and spine injuries, stroke, brain infections, and CSF disorders. Through 16 projects across 24 countries, the group aims to map care, improve treatment, foster innovation, and build local capacity. Emphasizing collaboration with nurses, allied health professionals, and patients, the research is tailored to local contexts
Northwest SCHOOL OF MEDICINE	**Northwest School of Medicine**	The Northwest School of Medicine, Pakistan, provides competency-based exceptional medical education and training to future healthcare professionals based on the biopsychosocial model with emphasis on research and innovation to ensure cost-effective, needs-based, value-based, community-oriented and patient-centered care.ISSI
ASBN	**American Society of Black Neurosurgeons (ASBN)**	The American Society of Black Neurosurgeons (ASBN) is a dynamic network of trailblazers committed to transforming the landscape of neurosurgical care. As a leading organization dedicated to fostering diversity, equity, and inclusion in the field, we empower Black neurosurgeons at every stage of their careers— from aspiring medical students to esteemed faculty members. The society exists to bridge opportunity gaps, amplify voices, and build a future where Black neurosurgeons thrive, innovate, and lead with impact.
Headway the brain injury association	**Headway: the brain injury association**	Headway is a UK-wide brain injury charity that provides support, services, and information to brain injury survivors, their families, and carers. The charity aims to improve life after brain injury, increase awareness and understanding of brain injury, and reduce its incidence. Headway achieves this through a network of local groups and branches, offering a range of services and activities.

Brain Capital Alliance	**Brain Capital Alliance**	Following on from the success of the OECD Neuroscience-inspired Policy Initiative (NIPI), the Brain Capital Alliance is an expanded, multi-national and multi-organisational programme. The programme focuses on neuroscience-inspired investment and public policy innovation as the two most powerful levers for change. It brings together radically diverse stakeholders spanning fields from brain science to policy, economics and finance. The Alliance brings together world-class contributors from various backgrounds to explore approaches to building Brain Capital at societal scale.
Northwest General Hospital & Research Centre	**Northwest General Hospital & Research Centre**	Northwest General Hospital and Research Centre is a 500+ Bed, State-of-the-Art Tertiary Care Hospital, Teaching Institute and a Research Centre, catering to the healthcare needs of the Khyber Pakhtunkhwa Region and rest of Pakistan, as well as Afghanistan and neighbouring countries.
INTS INTERNATIONAL NEUROTRAUMA SOCIETY	**International Neurotrauma Society (INTS)**	The INTS is a body of scientists and clinicians who aim to further brain and spinal cord injury research and to improve care for patients with these diseases. Its purpose is to foster the worldwide dissemination of Neurotrauma research and to supervise International Neurotrauma symposia throughout the world whilst preserving as best as possible, parity between brain and spinal cord injury research, global representation, gender balance and emphasis on both basic science and clinical research.
InTBIR International Initiative for Traumatic Brain Injury Research	**International Initiative for Traumatic Brain Injury Research (InTBIR)**	InTBIR is a cooperative effort of the European Commission (EC), the Canadian Institutes of Health Research (CIHR) and the National Institutes of Health (NIH) to coordinate and leverage clinical research activities on traumatic brain injury (TBI) research. InTBIR's goal is to improve health care and lessen the global burden of TBI by 2020 through the discovery of causal relationships between treatments and clinically meaningful outcomes. InTBIR seeks to encourage well-designed, hypothesis-driven studies that include the collection of high quality data followed by rigorous statistical analysis.
WORLD FEDERATION OF NEUROSURGICAL SOCIETIES 19 55	**World Federation of Neurosurgical Societies (WFNS)**	The World Federation of Neurosurgical Societies (WFNS) is a professional, scientific, non-governmental organization founded in 1955. Its primary goal is to improve neurosurgical care, training, and research globally, benefiting patients. The WFNS comprises 130 member societies, including 5 Continental Associations, 119 National Neurosurgical Societies, and 6 Affiliate Societies, representing over 30,000 neurosurgeons worldwide.

	**Mercy Ships**	Mercy Ships is an international, faith-based, non-governmental organization that deploys hospital ships to deliver free, high-quality healthcare to people in need, particularly in developing countries. Their mission is to provide hope and healing, improve healthcare, and meet the need for safe surgery worldwide, focusing on underserved populations who lack access to essential medical services.
	**The Global Alliance for Prevention of Spina Bifida (GAPSBiF)**	GAPSBiF, or the Global Alliance for the Prevention of Spina Bifida, is a multidisciplinary coalition advocating for mandatory folic acid fortification of staple foods globally to prevent neural tube defects like spina bifida. This coalition includes neurosurgeons, pediatricians, geneticists, epidemiologists, food scientists, and fortification policy experts, working together to promote public health interventions.
	**Brain Injury Canada (Lesion Cérébrale Canada)**	Brain Injury Canada is a national charitable organization that supports individuals living with acquired brain injury, their families, and caregivers. It provides education, awareness, and advocacy for the brain injury community, offering resources and information through its website. The organization works to improve access to services and supports for those affected by brain injury
	**Brain Trauma Foundation (BTF)**	The Brain Trauma Foundation (BTF) was founded in 1986 to develop research on traumatic brain injury (TBI). Since its formation the foundation's mission has expanded to improving the outcome of TBI patients nationwide through working to implement evidence-based guidelines for prehospital and in-hospital care, quality-improvement programs, and coordinating educational programs for medical professionals.
	**Society of British Neurological Surgeons (SBNS)**	The purpose of the SBNS is the study and advancement of Neurosurgery. This is achieved through the scientific meetings of the Society, through publications arising from deliberations about aspects of Neurosurgical practice and delivery of Neurosurgical services, through fostering professional relationships amongst neurosurgeons and through engagement with the public and bodies involved in the provision of care to patients requiring Neurosurgery.
	**Canadian Traumatic Brain Injury Research Consortium (CTRC)**	The CTRC was created to enhance collaborations amongst Canadian scientists working on different aspects of the continuum of care for traumatic brain injury patients, i.e. including prevention strategies, caring for patients in the critical phases following their accident, up to ensuring their continued rehabilitation and long-term optimal physical and psychological care. To do so, we encourage communication and collaboration between not only

		adult and pediatric scientists working on similar objectives, but also scientists working in basic, translational and clinical sciences. This synergistic cooperation will contribute to position Canada as one of the world leaders in traumatic brain injury.
	**Global Neuroscience Nurses**	Global Neuroscience nurses working in a comprehensive neuroscience nursing curriculum with the plan of training regional champions to maintain sustainability and scalability. Not only teaching skills but also research I, advocacy and leadership skills.
	**Foundation for International Education in Neurological Surgery (FIENS)**	FIENS' mission is to improve care of individuals with neurological disease throughout the world by support of education in neurosurgery, particularly via partnerships between established and emerging neurosurgical programs. FIENS provides hands-on training and education to neurosurgeons around the world. FIENS' volunteer neurosurgeons and personnel spend months on-site, teaching techniques to local neurosurgeons and setting up neurosurgery residency programs.
	**CENTER-TBI**	CENTER-TBI is a large European project that aims to improve the care for patients with Traumatic Brain Injury (TBI). CENTER-TBI brings the newest technologies and many of the world's leading TBI experts together in a much needed effort to tackle the silent epidemic of TBI. International and multidisciplinary collaboration are key elements to the project in which past dogmas will be left behind and innovative approaches undertaken.
	**ThinkFirst**	ThinkFirst's Mission is to prevent brain, spinal cord, and other injuries through education, research, and advocacy. Our goal is to foster a culture of safety and to positively influence behaviors thus reducing the occurrence of injuries. We believe that everyone should have the knowledge and resources necessary to protect themselves and others. Together, we can make a significant impact on reducing brain and spinal cord injuries.
	**Harvard Medical School Program in Global Surgery and Social Change (PGSSC)**	The Program in Global Surgery and Social Change (PGSSC) at Harvard Medical School, in partnership with Harvard Teaching Hospitals, Boston Children's Hospital, and Partners In Health (PIH), aims to improve global access to safe and affordable surgical and anesthesia care. It achieves this through research, advocacy, implementation science, and the training of future leaders in global surgical and health systems.
	**Intersurgeon**	InterSurgeon is an interactive website designed to bring individuals and organisations together in partnerships to improve surgical care globally.<br>It is committed to fostering collaborative partnerships in the field of Global Surgery that will advance clinical care, teaching, training, research and the

		provision and maintenance of medical equipment. Our dynamic virtual mapping platform allows stakeholders to interact within the global surgical ecosystem: providing insights into existing activities, gaps in services, and partnerships opportunities, while facilitating immediate connectivity and concrete action.
brain injury AUSTRALIA	**Brain Injury Australia**	Brain Injury Australia (BIA) is the central clearinghouse of information and gateway to nationwide referral for optimising the social and economic participation of all Australians living with brain injury. BIA was formed at the first national community-based conference on acquired brain injury (ABI) in 1986. The Federal Government began funding BIA in 1991.
	**Mission Brain**	A 501(c)(3) organization dedicated to expanding access to neurosurgical care worldwide. Throguh innovative training programs, research groups and global partnerships, it aims to address the critical shortage of neurosurgeons in underserved regions.
BRAIN INJURY ASSOCIATION OF AMERICA	**Brain Injury Association of America (BIAA)**	The Brain Injury Association of America (BIAA) is the voice of brain injury. They are dedicated to advancing awareness, research, treatment, and education and to improving the quality of life for all individuals impacted by brain injury. Through advocacy, they bring help, hope, and healing to millions of individuals living with brain injury, family caregivers, and professional clinicians.
UKABIF The United Kingdom Acquired Brain Injury Forum	**United Kingdom Acquired Brain Injury Forum (UKABIF)**	UKABIF raises awareness of acquired brain injury in parliament and amongst policy makers. Our primary objective is to ensure that people with ABI have early<br>access to local, specialist neurorehabilitation and follow up services in the community.
ACQUIRED BRAIN INJURY IRELAND	**Acquired Brain Injury, Ireland**	19,000 people in Ireland acquire a brain injury each year. At Acquired Brain Injury Ireland, through our community-based rehabilitation services, we help as many of them as we can to return to leading independent lives. We also provide information, training and practical help to those living with an acquired brain injury, as well as their carers and healthcare professionals.

INTERNATIONAL HEALTH SYSTEMS	**International Health Systems**	The International Health Systems group is an interdisciplinary team of academic engineers and clinicians. We research the improvement of healthcare, through systems thinking, with partners across the world. We believe in a model of participatory action research, based on equitable relationships.
LIFE+LIMB Primary Trauma Care	**Life+Limb / the Primary Trauma Care Foundation**	Life+Limb / the Primary Trauma Care Foundation has been involved in educating healthcare professionals for 30 years in over 90 low resource areas of the world to initially manage people who sustain major injury, including head injury.
BRITISH ASSOCIATION of NEUROSCIENCE NURSES www.bann.org.uk	**British Association of Neuroscience Nurses (BANN)**	BANN provides a voice for neuroscience nurses working within varying clinical and community settings.<br>Our main aims are to promote the highest standards of care within the speciality, to encourage and give the opportunity for exchange of ideas in this country and abroad and to encourage interest and awareness of the special needs of neuroscience patients.
ACRM AMERICAN CONGRESS OF REHABILITATION MEDICINE	**American Congress of Rehabilitation Medicine (ACRM)**	ACRM is a vibrant group with diverse individual backgrounds from all over the world – all united with the common interests in rehabilitation and evidence-based research to enhance the lives of those with disabling conditions, with a mission to improve the lives of people of all abilities through education, research, and advocacy to enable interdisciplinary, science-based, and person-centered rehabilitation
